# Laser-Promoted Immobilization of Ag Nanoparticles: Effect of Surface Morphology of Poly(ethylene terephthalate)

**DOI:** 10.3390/nano12050792

**Published:** 2022-02-26

**Authors:** Jakub Siegel, Daniel Grossberger, Jana Pryjmaková, Miroslav Šlouf, Václav Švorčík

**Affiliations:** 1 Department of Solid State Engineering, University of Chemistry and Technology Prague, 166 28 Prague, Czech Republic; daniel.grossberger@vscht.cz (D.G.); jana.pryjmakova@vscht.cz (J.P.); vaclav.svorcik@vscht.cz (V.Š.); 2 Institute of Macromolecular Chemistry, Academy of Sciences of the Czech Republic, Heyrovského nám. 2, 162 06 Prague, Czech Republic; slouf@imc.cas.cz

**Keywords:** laser processing, silver nanoparticles, polyethyleneterephthalate, ripple structures, surface morphology

## Abstract

In the last two decades, the importance of nanomaterials in modern technologies has been unquestionable. Metal nanoparticles are frequently used in many areas of science and technology, delivering unprecedented improvements to properties of the conventional materials. This work introduces an effective tool for preparing a highly enriched poly (ethylene terephthalate) (PET) surface with silver nanoparticles, firmly immobilized in the same surface area on polymer. We showed that besides pristine polymer, this approach may be successfully applied also on laser pre-treated PET with laser-induced periodic surface structures. At the same time, its final nanostructure may be effectively controlled by laser fluence applied during the immobilization process.

## 1. Introduction

Nanoparticles in a polymer matrix play an important role in the potential use of polymer composites in modern nanotechnologies due to their unique electrical [1], catalytic [2], mechanical [3], optical [4], and antimicrobial properties [5,6]. These unique properties are achieved by a combination of physicochemical properties of nanostructured materials [7,8] as well as by the specific surface area of the resulting composite [9], which ensures the yield in processes under consideration.

Silver nanoparticles (AgNPs) are widely used for their bacteriostatic or antimicrobial properties [5]. The antibacterial effects of AgNPs have been demonstrated on many bacteria, including bacteria resistant to common antibiotics [10]. They prevent the adhesion and proliferation of bacteria to the surface of materials and suppress microorganisms already adhered [6]. This property is used in the medical field where these materials protect equipment prone to biofilm formation, such as catheters and implants [11]. AgNPs are also used in the treatment of wounds, where they accelerate the conversion of fibroblasts into myofibroblasts (collagen-producing connective tissue cells) [12].

Catalysis is another important application area where metal nanoparticles play a crucial role [13,14]. Especially in the chemical industry, catalysis has become a key part of the production of chemicals by reducing the activation energy of reactions and accelerating their course [15]. The development of nanomaterials has contributed to heterogeneous catalysts with a large surface area [16]. Silver nanoparticles provide excellent results in dye reduction and removal [17]. Iqbal et al. [18] developed a hydrogel containing AgNPs capable of catalytically decomposing various types of azo dyes into less toxic products. Gels with immobilized AgNPs also serve to reduce amino groups. Begum et al. [19] created gels with AgNPs that remained functional for several months and contributed to the rapid reduction of 4-nitrophenol to 4-aminophenol in an aqueous medium with NaBH_4_.

In this work, we demonstrate that polarized light from a KrF excimer laser effectively mediates the immobilization process of silver NPs from their colloid solution. As a result, PET foils are evenly decorated with Ag NPs regardless of the specific surface morphology of polymer entering the immobilization process. Thus, our approach enables effective control resulting in the surface morphology of AgNPs/polymer interface at nanoscale. We believe that our AgNPs/PET composites may find its application as novel antimicrobial surfaces for tissue engineering.

## 2. Experimental

### 2.1. Materials, Apparatus and Procedures

Silver nanoparticles were prepared by electrochemical dissolution of two Ag electrodes in sodium citrate electrolyte according to the procedure described in [20]. After the synthesis the concentration of AgNPs colloid was determined by AAS. For further experiments, concentration of AgNPs was set to 30 mg L^−1^ by adding 1 mM solution of sodium citrate. Before the immobilization process, AgNPs colloids were characterized by TEM.

The immobilization process was carried out using KrF excimer laser (COMPex Pro 50F, Coherent, Inc., Silicon Valley, CA, USA, wavelength 248 nm, pulse duration 20–40 ns, repetition rate 10 Hz, 6000 pulses). PET foil (Goodfellow Ltd., Cambridge, UK, thickness 50 µm) was centered in the spectroscopic cuvette (Starna Scientific Ltd., Ilford, UK, type 3/Q/100) and charged with the solution of AgNPs. In this set-up, PET foil was irradiated by 6000 laser pulses at fluencies 10 and 24 mJ cm^−2^ through a linear polarizer (UV-grade fused silica prism, model PBSO-248-100). In our experiments, as-received (pristine) PET and laser pre-treated PET foils (with LIPSS structure) were used. Laser pre-treatment was carried out on the same KrF laser using the laser fluence of 7 mJ cm^−2^ according to procedure described in [21]. The resulting surface morphology of PET exhibited the coherent ripple structure known as LIPSS (laser-induced periodic surface structure).

### 2.2. Analytical Methods

UV–Vis absorption measurements were carried out on a Perkin Elmer Lambda 25 UV–Vis spectrophotometer (PerkinElmer Inc., Waltham, MA, USA, deuterium and halogen lamp light sources, range 350–800 nm, room temperature) equipped with module for the measuring of solid samples. The scanning speed was set to 240 nm min^−1^ with a data collection interval of 1 nm.

The concentration of Ag in colloidal solutions was determined by atomic absorption spectrometry (AAS) with a flame atomization technique. Measurements were performed on a Varian AA880 device (Varian Inc., Palo Alto, CA, USA). The typical uncertainty of concentration determined by this method was less than 3%.

TEM characterization of AgNPs colloids was performed using JEOL JEM-1010 (JEOL Ltd., Akishima, Japan) operated at 80 kV. Particle size was measured from the TEM micrographs and calculated by taking at least 500 particles into account, using AnalysSIS 2.0 software. Samples for TEM were centrifuged, and NPs transferred into distilled water. Drop of colloidal solution was placed on a copper grid coated with a thin amorphous carbon film on filter paper. Samples were air-dried and kept under vacuum in a desiccator before placing on a specimen holder.

AFM analysis was performed on Dimension ICON (Bruker Corp., Billerica, MA, USA) in ScanAsyst tapping mode in the air. The measurement was performed with an antimony doped silicon probe-type RTESPA-150. The NanoScope Analysis software was used to evaluate the data.

The chemical composition of the surface layer was determined by the XPS method using Omicron Nanotechology ESCAProbeP spectrometer (Omicron Nanotechnology GmbH, Taunusstein, Germany). The X-ray source monochromated at 1486.7 eV with the step size of 0.05 eV was used. The spectra evaluation was carried out by CasaXPS software. The uncertainty of the measurement was less than 3%.

Surface visualization of samples with immobilized AgNPs was accomplished on a high-resolution FEGSEM microscope MAIA3 (TESCAN, Brno, Czech Republic) equipped with detectors for secondary and backscattered electrons. Measurements were performed in high-resolution mode at an accelerating voltage of 3 kV.

## 3. Results and Discussion

**Figure 1:**
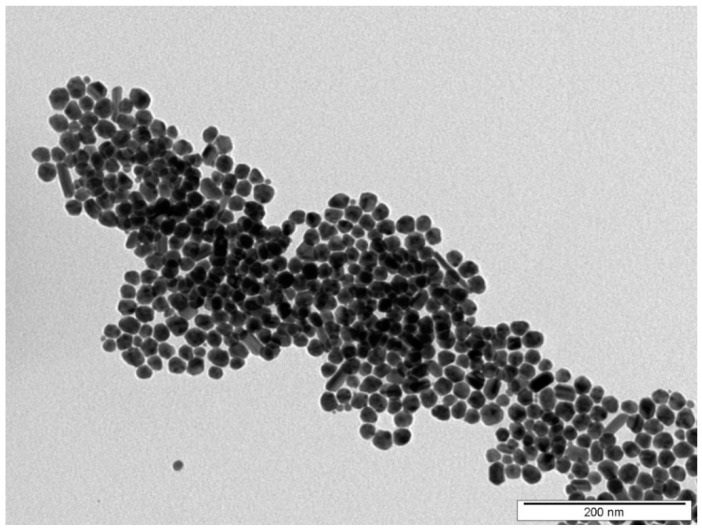
TEM image of synthesized AgNPs used in immobilization process.

The shape and the morphology of prepared nanoparticles were characterized by TEM. Figure 1 shows that synthetized silver nanoparticles were mostly spherical with a minimal incidence of rods. The average size of spherical AgNPs was around 20 nm.

**Figure 2:**
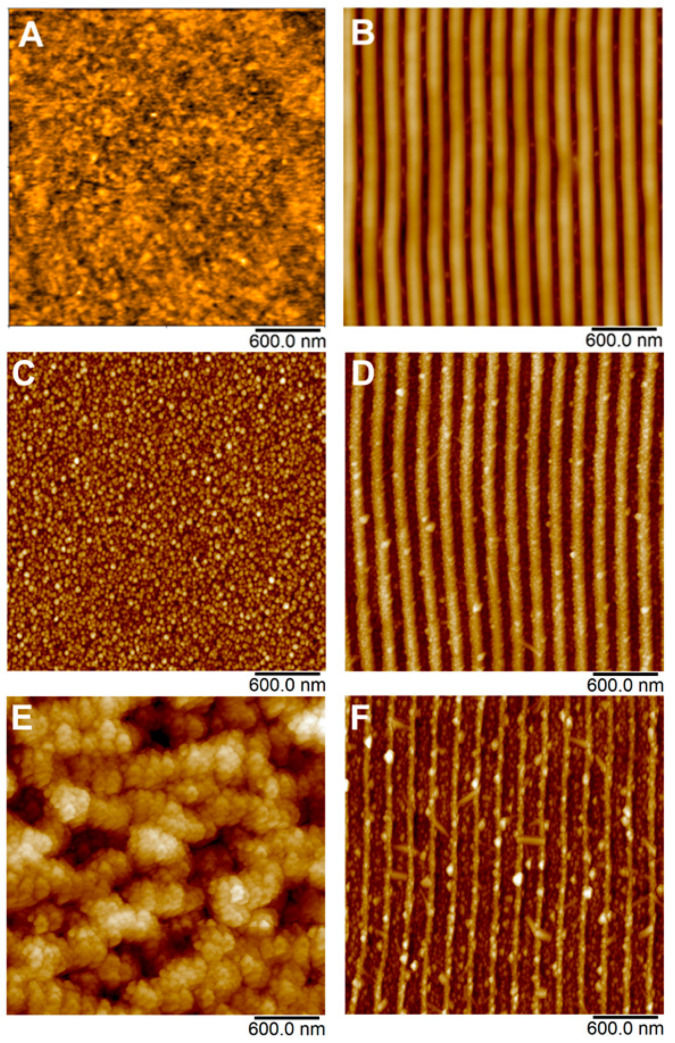
AFM images of pristine PET (**A**); laser pre-treated PET with LIPSS structure (**B**); and PET with immobilized AgNPs on pristine PET (**C**,**E**) and PET with LIPSS structure (**D**,**F**).

The surface morphology of pristine PET, laser pre-treated PET with LIPSS structure and PET with immobilized silver NPs was measured by AFM and is depicted in Figure 2. Related surface parameters derived from AFM measurements such as surface roughness *R*_a_ and surface area difference SAD (the ratio of the difference between the measured area and the scanned area to the scanned area) are summarized in Table 1. It is evident from Figure 2A that pristine PET exhibited pretty flat surface morphology, which turned into coherent ripple patterns once the PET was irradiated with laser light (Figure 2B). These structures are referred to as LIPSS [8]. 

It is evident from Table 1 that once the LIPSS were formed, both surface roughness and SAD increased significantly compared to new PET, which is typical for rugged surfaces with complicated morphology [22]. Figure 2C–F shows the development of surface morphology of PET after immobilization of silver nanoparticles from AgNPs colloids. In addition to the specific morphology changes of the underlying polymer, which will be discussed in detail below, these AFM images clearly show that the polymer surface was evenly decorated by AgNPs, regardless of particular fluence applied or polymer pre-treatment (pristine or LIPSS). Obviously, lower immobilization fluence did not cause any visible changes in the morphology of underlying PET in the case of both pristine and laser-modified samples (Figure 2C,D), which was quantified by a similar *R*_a_ and SAD, especially in the case of LIPSS/PET and AgNPs/LIPSS/PET samples (see Table 1). However, when higher laser fluence was applied, the surface morphology of pristine PET was dramatically changed (worm-like structure appeared, accompanied by a significant increase of *R*_a_ and SAD, see Table 1) due to considerable material ablation. In the case of AgNPs immobilization on PET with LIPSS, higher fluence degraded the structures in terms of their narrowing; however, the LIPSS were preserved.

A significant difference between the effect of higher fluence applied on pristine PET a PET with LIPSS in the view of the change of surface morphology may be attributed to the reorganization of macromolecular domains and the recrystallization of the polymer typical in the process of LIPSS formation [21,22]. As a result, considerably higher energy is needed to perturb the organized structure of the LIPSS than in the case of pristine PET. Therefore, the observed changes in morphology during the immobilization process at elevated laser fluence (24 mJ cm^−2^) are much more pronounced in the case of pristine PET when compared to PET with LIPSS structure.

**Figure 3:**
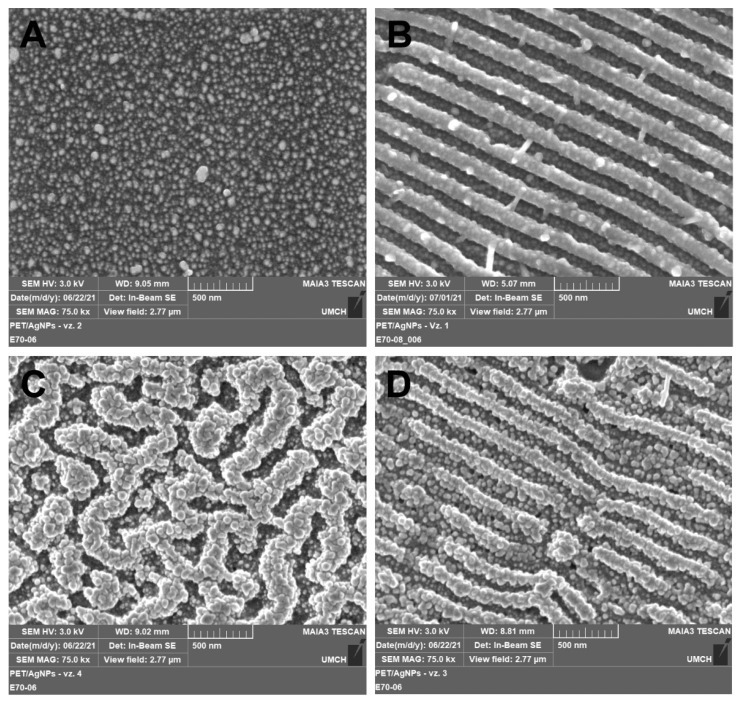
FEGSEM micrographs showing the surface morphology of PET immobilized with AgNPs at laser fluencies of 10 (**A**,**B**) and 24 mJ cm^−2^ (**C**,**D**) on pristine PET (**A**,**C**) and PET with LIPSS structure (**B**,**D**). Micrographs were recorded with an in-beam SE detector at accelerating voltage 3 kV.

The surface morphology of AgNPs decorated polymers was observed also by FEGSEM microscopy (Figure 3) to better visualize the AgNPs through the different material contrast (metal/polymer). Figure 3A,B show smooth and coherently patterned PET (LIPSS) with immobilized Ag nanoparticles under the fluencies of 10 mJ cm^−2^. Similarly to AFM analysis, it follows from SEM micrographs that under the lower immobilization fluence, the morphology of the underlying polymer remained fully preserved and AgNPs evenly decorated the polymer. When the immobilization fluence increased to 24 mJ cm^−2^, the most pronounced change in the surface morphology occurred at originally planar polymer, while PET with LIPSS structure exhibited only minor disturbances of the periodic structure (see Figure 3C,D). The cause of this different behavior was discussed in the previous paragraph and most probably was related to the presence of the LIPSS themselves. It is also evident that regardless of the nature of the polymer surface after nanoparticle immobilization (smooth, rough, or with LIPSS structure), the AgNPs were always located at the same surface area of the polymer.

To track the changes in the chemical composition of the polymer surface, we performed the XPS analysis. Atomic concentrations of elements are summarized in Table 2. Atomic concentrations of C and O in pristine PET correspond well with the theoretical content based on polymer stoichiometry [23], 71.4 and 28.6 for C and O, respectively. Once the LIPSS structures were formed, the elemental composition was shifted towards oxygen at the expense of carbon, the effect of which was due to rearrangement of polymer macromolecules, their reorientation, and partial oxidation during the process of ripple patterns formation [22]. After the AgNPs immobilization, Ag was detected on all samples in concentrations exceeding 10 at.%, regardless on both specific laser fluence applied and input polymer treatment; however, one can see slightly increased Ag content on PET with LIPSS structure. This may probably be caused by the increased affinity of silver nanoparticles (which usually carry positive charge [24]) to the LIPSS surface, which exhibits a higher concentration of oxygen than pristine PET and thus carries a partial negative charge. It is evident that the higher the immobilization fluence, the higher the Ag content, regardless of whether or not the LIPSS structure is present.

**Figure 4:**
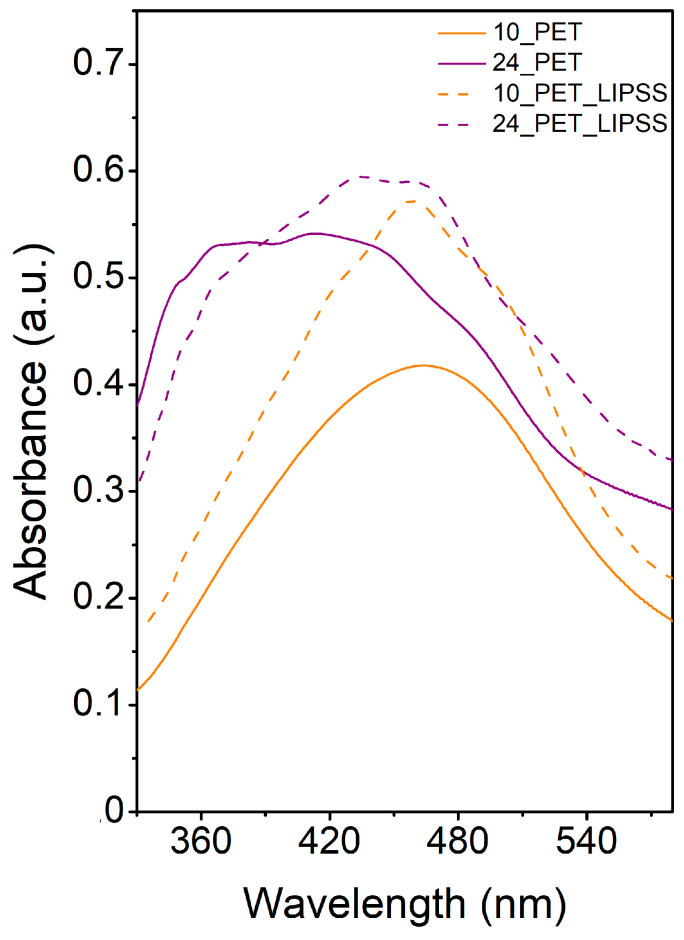
UV-Vis absorption spectra of AgNPs immobilized with the fluence of 10 mJ cm^−2^ on pristine PET (10_PET) and PET with LIPSS structure (10_PET_LIPSS) and with the fluence of 24 mJ cm^−2^ on pristine PET (24_PET) and PET with LIPSS structure (24_PET_LIPSS).

In Figure 4, the absorption spectra of the samples after irradiation in a colloidal solution of AgNPs are shown. We present the data in the range of wavelengths from 330 to 580 nm because the maximum of the LSPR band of silver nanoparticles is located in this area (typically 400–410 nm [25]). The coalescence of NPs did not cause the extension of the absorbance bands during immobilization into the PET surface (see SEM images, Figure 3), but rather by the change of dielectric environment, the essence of which is the shortening of polymer chains, the formation of volatile substances and their crosslinking, the formation of carbon clusters, and in some cases, the formation of multiple bonds [26]. 

However, the change of dielectric conditions is not the only phenomenon observed in the spectra. The multimodality of the absorption bands in the samples with LIPSS structure was caused by the coupling effect [27]. The increase in absorbance is due to the excitation of the waveguide modes of the structure. These modes are related to the electromagnetic distribution inside the material; they oscillate inside the waveguide and partially penetrate the outer area. The rate of coupling size affects the refractive index, wavelength, and shorter structure size (width) [28]. The formation of LIPSS changed the refractive index on the PET surface [29]. The change in the refractive index likely manifested itself also in the sample with a worm-like structure (see curve 24_PET). Coupling was observed in the spectra of these samples as small peaks at 350, 370, 430, 460 nm. LSPR, therefore, influenced the measured UV-Vis spectra in silver nanoparticles (410 nm), the change of dielectric environment due to polymer modification, and electromagnetic coupling. Generally, the higher absorbance of the structures formed with the fluence of 24 mJ cm^−2^ (compared to their 10 mJ cm^−2^ counterparts) is in accordance with the increase of multiple double bonds as a consequence of cross-linking of macromolecules after the interaction of intense light with the polymer.

## 4. Conclusions

We successfully immobilized silver nanoparticles on the PET surface via an excimer laser-mediated approach. We demonstrated the possibility to decorate both pristine and laser pre-treated PET exhibiting flat or coherently patterned surface structure (LIPSS). AFM and TEM analyses showed that regardless of the surface morphology of the polymers entering the immobilization process, the PET is evenly decorated by AgNPs with a surface concentration of Ag exceeding 10 at.%. By the variation in the immobilization laser fluence, one can effectively change the resulting morphology from originally flat or LIPSS structure to worm-like or perturbed LIPSS structure in case of 10 and 22 mJ cm^−2^, respectively. We believe that our highly AgNPs enriched polymeric composites may find applications in tissue engineering as novel antimicrobial biomaterials.

## Figures and Tables

**Table 1:** Image characteristics (average surface roughness *R*_a_ and surface area difference SAD) derived from AFM analysis. The designation of the samples corresponds to the Figure 2.

Sample	Laser Fluence (mJ cm^−2^)	*R*_a_ (nm)	SAD (%)
PET (A)	-	0.78	4.93
LIPSS/PET (B)	-	10.5	27.2
AgNPs/PET (C)	10	4.59	16.1
AgNPs/PET (D)	24	27.5	26.2
AgNPs/LIPSS/PET (E)	10	10.3	26.7
AgNPs/LIPSS/PET (F)	24	20.9	32.9

**Table 2:** Concentrations of silver Ag3d, carbon C1s, and oxygen O1s (in at.%) in pristine, LIPSS modified, and AgNPs immobilized PET, derived from XPS analysis. The designation of the samples corresponds to Figure 2.

Sample	Laser Fluence (mJ cm^−2^)	Ag	C	O
PET (A)	-	-	71.0	29.0
LIPSS/PET (B)	-	-	67.2	32.8
AgNPs/PET (C)	10	10.7	67.7	21.6
AgNPs/PET (D)	24	11.3	66.5	22.2
AgNPs/LIPSS/PET (E)	10	13.1	65.8	21.1
AgNPs/LIPSS/PET (F)	24	15.1	63.5	21.4

## Data Availability

The data presented in this study are available on request from the corresponding author.
